# Supplementary material for: Exploring the Biocontrol Capability of Non-Mycotoxigenic Strains of Penicillium expansum
Source: Toxins (Basel). 2024 Jan 17;16(1):52. doi: 10.3390/toxins16010052 (PMC10820982; doi:10.3390/toxins16010052)
Supplement: Supplementary file 1 [file toxins-16-00052-s001.zip › toxins-2787556-supplementary.pdf]

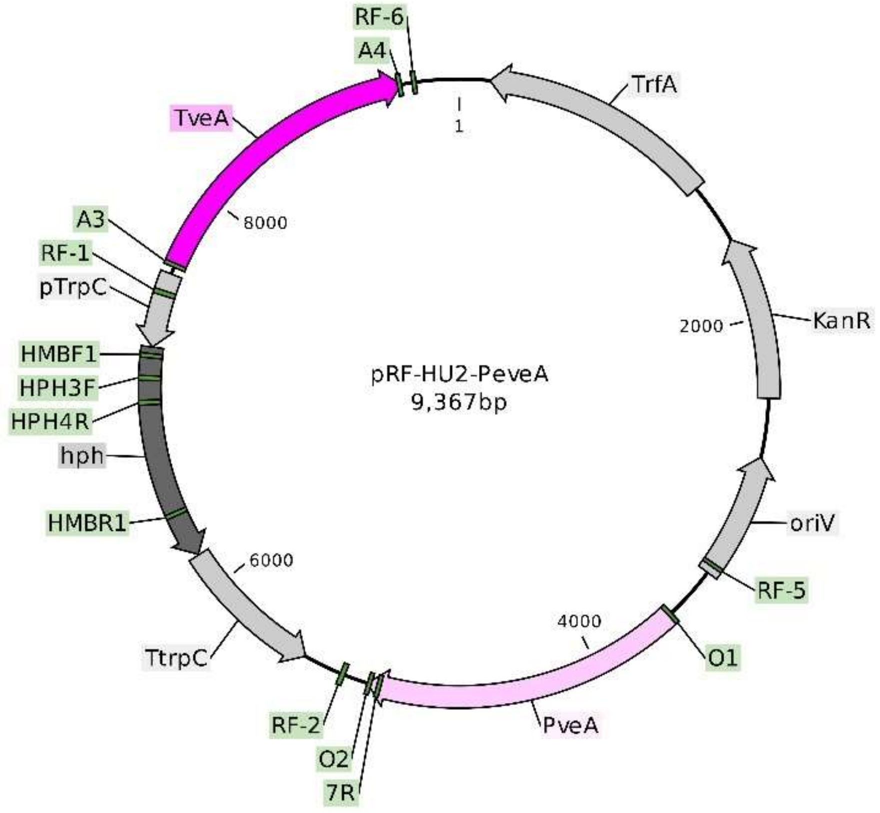


**Figure S1**. Physical map of plasmid pRFHU2-*veA*.

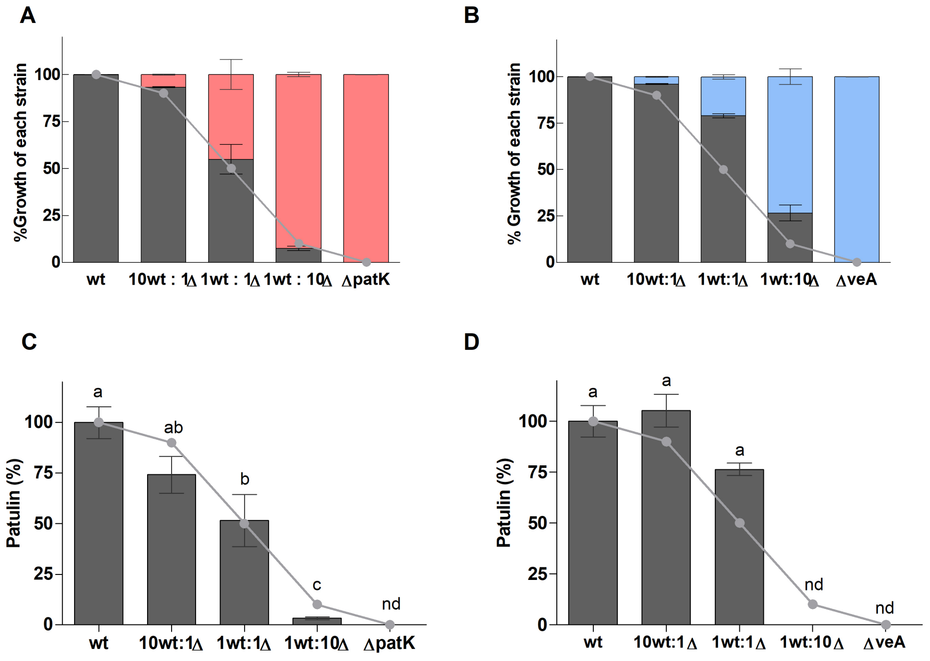


**Figure S2.** Competitiveness of ΔpatK (A) (red bars) and ΔveA (B) (blue bars) knockout mutants against the patulin producer P. expansum (grey bars) on PDA after 4 days of incubation. Competitive assays were conducted at ratios of 10:0, 10:1, 1:1, 1:10, and 0:10 (WT vs. Δ). The percentage of each strain at 4 days post-inoculation was determined using qPCR. The expected values for the WT strain are represented by gray lines. Values represent the mean ± the standard error of the mean of at least three biological replicates. Statistical analyses are presented in Table S3. Patulin production at 4 days post-inoculation during competition assays of WT vs. Δ*patK* (C) and Δ*veA* (D) knockout mutants analyzed by HPLC. Letters in the same panel show significant differences (one-way ANOVA and Tukey's HSD test, $p < 0.05$). nd—not detected during the testing conditions.

**Table S1.** List of primers used in this study.

| *Name* | *Sequence (5′ → 3′)* | *Description* |
|---|---|---|
| **PeveA mutant** | | |
| **veA_O1** | GGTCTTAAUTTGACGTGGCTTCCAATACA | Amplification of upstream region |
| **veA_O2** | GGCATTAAUGGTGGTCTGTTTGCCATTTT | Amplification of upstream region |
| **veA_A3** | GGACTTAAUCACAATGGCGCAAAATACAC | Amplification of downstream region |
| **veA_A4** | GGGTTTAAUATCATGGGCTCCGTAATCAG | Amplification of downstream region |
| **veA-1F** | ACAGCACAACTCCTCCAAGG | Screening gene deletion; T-DNA copy number |
| **veA-2R** | TAGGGCTGGAGTAGCCTCAA | Screening gene deletion |
| **veA-6R** | GGCACGCTTGTATGCTAACT | Screening gene deletion and qPCR (*in vitro* competition assay) – screening gene of interest |
| **veA – 7R** | TATTTTGCCGGTCGCAGTAT | T-DNA copy number |
| **veA-8F** | CCGGCTGTATCGCAAGGTAT | Screening gene deletion |
| **veA-5F** | TGAAGATACTTTCGGCCTTG | qPCR (*in vitro* competition assay) – screening gene of interest |
| **Other primers** | | |
| **RF-1** | AAATTTTGTGCTCACCGCCTGGAC | *E. coli* colonies selection and DNA sequencing |
| **RF-2** | TCTCCTTGCATGCACCATTCCTTG | *E. coli* colonies selection and DNA sequencing |
| **RF-5** | GTTTGCAGGGCCATAGAC | *E. coli* colonies selection and DNA sequencing |
| **RF-6** | ACGCCAGGGTTTTCCCAGTC | *E. coli* colonies selection and DNA sequencing |
| **HMBF1** | CTGTCGAGAAGTTTCTGATCG | Screening insertion resistant marker |
| **HMBR1** | CTGATAGAGTTGGTCAAGACC | Screening insertion-resistant marker |
| **HPH3F** | TATGTCCTGCGGGTAAATAGCTG | qPCR (*in vitro* competition assay) – screening mutants |
| **HPH4R** | GAGATGCAATAGGTCAGGCTCTC | qPCR (*in vitro* competition assay) – screening mutants |
| **PepatK_Li-F** | GACGCTGGGCTACTGGATTG | qPCR (*in vitro* competition assay) – screening gene of interest (*patK*) (1) |
| **PepatK_Li-R** | TCGTGCGTGAGGCCAGTAT | qPCR (*in vitro* competition assay) – screening gene of interest (*patK*) (1) |
| **Pe37-S** | GCTCTGGTCTACGACTCCTC | 37s ribosomal protein s24 as reference gene |
| **Pe37-R** | GGAAGCCTTCTCGATCTTGC | 37s ribosomal protein s24 as reference gene |
| **PeTub-1F** | AGCGGTGACAAGTACGTTCC | β-tubulin as protein as reference gene |
| **PeTub-2R** | ACCCTTGGCCCAGTTGTTAC | β-tubulin as protein as reference gene |

[1]Li, B., Chen, Y., Zong, Y., Shang, Y., Zhang, Z., Xu, X., Wang, X., Long, M., & Tian, S. Dissection of patulin biosynthesis, spatial control and regulation mechanism in Penicillium expansum. *Environ. Microbiol*. **2019**, *21(3)*, 1124–1139. https://doi.org/10.1111/1462-2920.14542 [50]

**Table S2**. Statistical analysis of the results presented in Figure 5. A one-way analysis of variance (ANOVA) with Tukey's honestly significant difference (HSD) procedure was performed. Similar letters in the same sample/ experiments indicate that there are no statistically significant differences ($p < 0.05$). nd: non detected.

| | **Figure 5A** | | **Figure 5B** | |
|---|---|---|---|---|
| | | | | |
| **COUNTING** | t0 WT vs Δ*patK* | | t0 WT vs Δ*veA* | |
| | **WT exp.1** | **WT exp.2** | **WT exp.1** | **WT exp.2** |
| **wt** | A | A | A | A |
| **10wt:1Δ** | A | A | A | A |
| **1wt:1Δ** | B | B | B | B |
| **1wt:10Δ** | B | C | C | B |
| **Δ** | nd | nd | nd | nd |
| | t0 WT vs Δ*patK* | | t0 WT vs Δ*veA* | |
| | **Δ*patK* exp.1** | **Δ*patK* exp.2** | **Δ*veA* exp.1** | **Δ*veA* exp.2** |
| **wt** | nd | nd | nd | nd |
| **10wt:1Δ** | B | C | C | B |
| **1wt:1Δ** | A | B | B | A |
| **1wt:10Δ** | A | A | A | A |
| **Δ** | A | A | A | A |

| | **Figure 5C** | | **Figure 5D** | |
|---|---|---|---|---|
| | | | | |
| **COUNTING** | t7 WT vs Δ*patK* | | t7 WT vs Δ*veA* | |
| | **WT exp.1** | **WT exp.2** | **WT exp.1** | **WT exp.2** |
| **wt** | A | A | A | A |
| **10wt:1Δ** | A | A | A | AB |
| **1wt:1Δ** | B | B | A | B |
| **1wt:10Δ** | C | C | B | C |
| **Δ** | nd | nd | nd | nd |
| | t7 WT vs Δ*patK* | | t7 WT vs Δ*veA* | t7 WT vs ΔpatK |
| | **Δ*patK* exp.1** | **Δ*patK* exp.2** | **Δ*veA* exp.1** | **Δ*veA* exp.2** |
| **wt** | nd | nd | nd | nd |
| **10wt:1Δ** | C | D | C | C |
| **1wt:1Δ** | B | C | C | C |
| **1wt:10Δ** | A | B | B | B |
| **Δ** | A | A | A | A |

| | **Figure 5E** | | **Figure 5F** | |
|---|---|---|---|---|
| | | | | |
| **qPCR** | t7 WT vs Δ*patK* | | t7 WT vs Δ*veA* | |
| | **WT exp.1** | **WT exp.2** | **WT exp.1** | **WT exp.2** |
| **wt** | A | A | A | A |
| **10wt:1Δ** | A | A | A | AB |
| **1wt:1Δ** | B | B | B | B |
| **1wt:10Δ** | C | C | C | C |
| **Δ** | nd | nd | nd | nd |
| | t7 WT vs Δ*patK* | | t7 WT vs Δ*veA* | t7 WT vs ΔpatK |
| | **Δ*patK* exp.1** | **Δ*patK* exp.2** | **Δ*veA* exp.1** | **Δ*veA* exp.2** |
| **wt** | nd | nd | nd | nd |
| **10wt:1Δ** | C | C | D | C |
| **1wt:1Δ** | B | B | C | C |
| **1wt:10Δ** | A | A | B | B |
| **Δ** | A | A | A | A |

**Table S3**. Statistical analysis of the results presented in Figure S2. A one-way analysis of variance (ANOVA) with Tukey's honestly significant difference (HSD) procedure was performed. Similar letters in the same sample/ experiments indicate that there are no statistically significant differences ($p < 0.05$). nd: non detected.

| | **Figure S2A** | **Figure S2B** |
|---|---|---|
| | | |
| **qPCR** | t4 WT vs Δ*patK* | t4 WT vs Δ*veA* |
| | **WT exp.1** | **WT exp.1** |
| **wt** | A | A |
| **10wt:1Δ** | A | A |
| **1wt:1Δ** | B | B |
| **1wt:10Δ** | C | C |
| **Δ** | nd | nd |
| | t4 WT vs Δ*patK* | t4 WT vs Δ*veA* |
| | **Δ*patK* exp.1** | **Δ*veA* exp.1** |
| **wt** | nd | nd |
| **10wt:1Δ** | C | D |
| **1wt:1Δ** | B | C |
| **1wt:10Δ** | A | B |
| **Δ** | A | A |
